# Supplementary material for: Type 1 diabetes and parasite infection: An exploratory study in NOD mice
Source: PLoS One. 2024 Oct 22;19(10):e0308868. doi: 10.1371/journal.pone.0308868 (PMC11495574; doi:10.1371/journal.pone.0308868)
Supplement: S1 Table — (PDF) [file pone.0308868.s001.pdf]

S1 Table: Sequences of Primers used for qRT-PCR

**REFERENCE GENES**	**Name**	**FORWARD**	**REVERSE**	**References**
hprt	*Hypoxanthine-guanine phosphoribosyl transferase*	ATTAGCGATGATGAACCAG	CTTGAGCACACAGAGGG	1
ywhaz	*Tyrosine 3-Monooxygenase/Tryptophan 5-Monooxygenase Activation Protein Zeta*	GTTACTTGGCCGAGGT	GGAGTTCAGGATCTCGT	2
**TARGET GENES**	**Name**	**FORWARD**	**REVERSE**	
spp1 (FL-*opn*)	*Secreted phosphoprotein 1*	TCTGATGAGACCGTCACTGC	CCTCAGTCCATAAGCCAAGC	1
i-*opn*	*intracellular-osteopontin*	GCCTGTTTGGCATTGCCTCCTC	CACAGCATTCTGTGGCGCAAGG	1
CD44	*CD44*	TATCTCCCGGACTGAGG	AGGCATTGAAGCAATATGT	1
il-1b	*Interleukin 1 beta*	AGGCAGGCAGTATCAC	CACACCAGCAGGTTATC	2
Tbet	*Th1-specific T-box transcription factor*	AACAAGGGGGCTTCCAAC	TGGCAAAGGGGTTGTTGT	this publication
STAT-1	*Signal transducer and activator of transcription 1*	TGGGTGCATTATGGGC	TTTCGTGTAGGGCTCC	this publication
IL-4	*Interleukin 4*	GGAGCCATATCCACGG	AAGCCCTACAGACGAG	3
IL-10	*Interleukin 10*	CCAAGCCTTATCGGAAATG	CCTGAGGGTCTTCAGC	3
IL-17	*Interleukin 17*	CTACCTCAACCGTTCCAC	GCATCTTCTCGACCCT	this publication
IFN-g	*Interferon gamma*	CTTCTTCAGCAACAGCAAGG	TGAGCTCATTGAATGCTTGG	3
iNOS-2	*(Exon 6) nitric oxide synthase 2, inducible*	ACAGGAACCTACCAGC	GGTTGGACCACTGGA	2
**LEISHMANIA GENE:** *SsrRNA*	*small subunit ribosomal RNA*	CCATGTCGGATTTGGT	CGAAACGGTAGCCTAGAG	**2**

References

1. Giraud, E., Rouault, E., Fiette, L. Colle, J-H, Smirlis and Melanitou, E. Osteopontin in the host response to *Leishmania amazonensis*. *BMC Microbiol* **19**, 32 (2019). https://doi.org/10.1186/s12866-019-1404-z
2. Giraud, E., Lestinova, T., Derrick, T., Martin, O., Dillon, R.J., Volf, P., et al. *Leishmania* proteophosphoglycans regurgitated from infected sand flies accelerate dermal wound repair and exacerbate leishmaniasis via insulin-like growth factor 1-dependent signalling. PLoS Pathog **14**(1) (2018): e1006794. https://doi.org/10.1371/journal.ppat.1006794
3. Giraud, E., Lecoeur, H., Rouault, E., Goyard, S., Milon, G., Lang, T. A combined luciferase-expressing Leishmania imaging/RT-qPCR assay provides new insights into the sequential bilateral Parasitology International, **63**(1) (2014). https://doi.org/10.1016/j.parint.2013.08.013
