# Supplementary material for: Type 1 diabetes and parasite infection: An exploratory study in NOD mice
Source: PLoS One. 2024 Oct 22;19(10):e0308868. doi: 10.1371/journal.pone.0308868 (PMC11495574; doi:10.1371/journal.pone.0308868)
Supplement: S2 Table — (PDF) [file pone.0308868.s002.pdf]

S2 Table: Type 1 diabetes in response to *L. amazonensis*

A: Representative T1D-related phenotypes

	**15-20 wks**	**21-25 wks**	**T1D >26 wks**	**Mortality >26 wks**
**NOD Non-INF**	2/10 (20%)	4/10 (40%)	6/10 (60%)	3/10 (30%)
**NOD + *Leish*.**	2/6 (33%)	5/6 (83.3%)	5/6 (83.3%	4/6 (67%)
**KO Non-INF**	11/20 (55%)	14/20 (70%)	16/20 (80%)	0/20 (0%)
**KO + *Leish*.**	4/12 (33.3%)	7/12 (58%)	8/12 (68%)	5/12 (41%)

B: Impact of *L. amazonensis* infection on T1D on wild-type and *opn* knockout NOD mice

	**T1D**		**INFECTION**
	**Non-INF**	**INF**	
**$NOD^{+/+}$**	Protection	++Acceleration	+Proliferation of parasites
**NOD.*OPN* $^{-/-}$**	Acceleration	+Acceleration	Non-proliferation
