# Supplementary material for: Type 1 diabetes and parasite infection: An exploratory study in NOD mice
Source: PLoS One. 2024 Oct 22;19(10):e0308868. doi: 10.1371/journal.pone.0308868 (PMC11495574; doi:10.1371/journal.pone.0308868)
Supplement: S3 Table — (PDF) [file pone.0308868.s003.pdf]

S3 Table: **Comparative statistical evaluation of parasite infectivity in BMF**. The statistical analysis as described in the Material section has as follows: "Mean parasite intensity" is the mean number of parasites per infected host cell. The non-infected cells are not taken into consideration. "Prevalence" is the percentage of infected cells and provides information on the relative sizes of the cells in the study (infected and uninfected)."Crowding" is a measure of the parasites' density and is defined as the sum of crowding values (parasites living in a cell) divided by the total number of parasites (Quantitative Parasitology (QP3.0) statistical software). NS: non-significant.

A. Parasitic infection NOD

**Host (BMF)**	**Mean Intensity N° (SD)**		**Mean Crowding**		**Host size (Infected Cell n°)**	
	**24 h *pi***	**48 h *pi***	**24 h *pi***	**48 h *pi***	**24 h *pi***	**48 h *pi***
**WT**	3.64 (2.29)	12.13 (8.8)	6.72	24.07	152 (117)	58 (54)
**KO**	8.21 (2.4)	8.5 (2.38)	9.35	8.76	29 (0)	8 (0)

B. Parasitic load statistics NOD. Statistics: Fisher's exact test 2-tailed

**Hosts**	**Prevalence (p-value)**	**Mean Intensity (p-value)**	**Mean Crowding**
**WT 24h *vs* 48h**	0.770 *vs* 0.931 (P=0.0054)	0.0010	6.72 *vs* 24.07<br>P<0.05, CI 97.5%
**KO 24h *vs* 48h**	1 *vs* 1 (P-value NS)	NS	9.35 *vs* 8.76<br>P>0.05 NS
**WT 24h *vs* KO 24h**	0.770 *vs* 1 (P=0.0011)	0.001	6.72 *vs* 9.35<br>P>0.05 NS
**WT 48h *vs* KO 48h**	0.931 *vs* 1(P-value NS)	NS	24.07 *vs* 8.76<br>P<0.05, CI 97.5%
