# Supplementary material for: Type 1 diabetes and parasite infection: An exploratory study in NOD mice
Source: PLoS One. 2024 Oct 22;19(10):e0308868. doi: 10.1371/journal.pone.0308868 (PMC11495574; doi:10.1371/journal.pone.0308868)
Supplement: S1 Fig — A. BMF cell markers and B. Dendritic cell markers. C. Summary showing similar characteristics of the BMF and DC between the NOD/LtJ (or NOD+/+) wild type and opn (NOD.opn-/-) knockout mice. The DC population was MHC-IIlow and mainly CD11c- with few clusters of CD11c+ containing cells. (PDF) [file pone.0308868.s004.pdf]

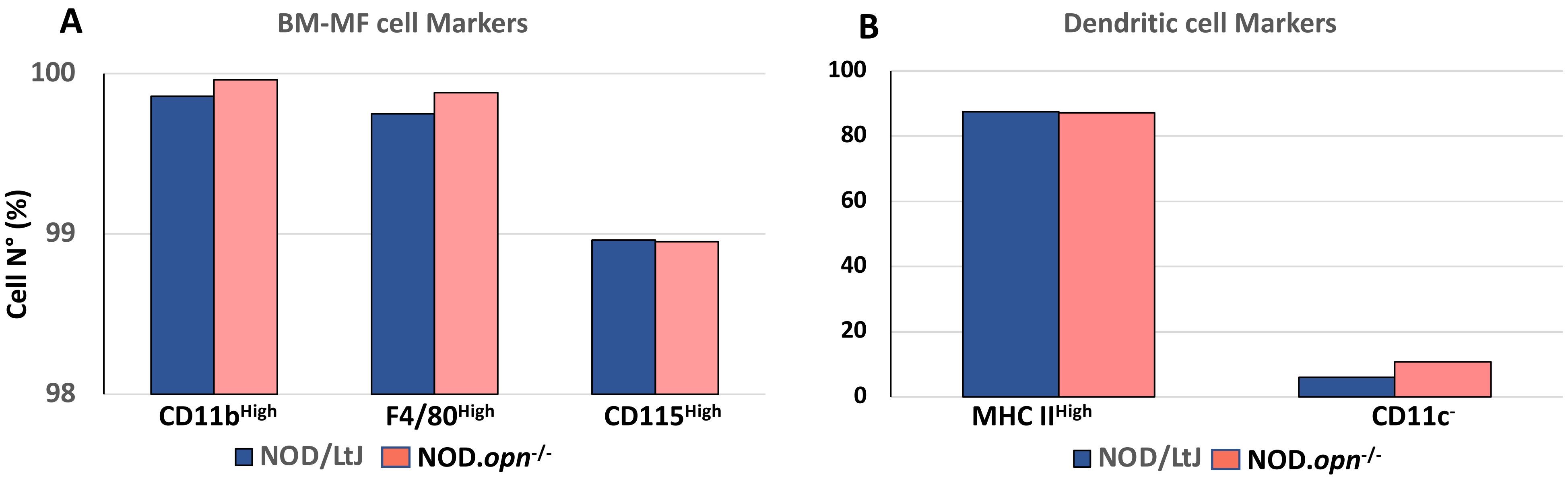

C

NOD/*Lt*J ($^{+/+}$) & NOD.*opn*^{KO}

BMF characterization: CD11b^{High} F4/80^{High}, CD115^{High}

DC characterization: CD11c^{+}, MHC-II^{low}

Mainly CD11c^{-} cell populations
with few clusters of CD11c^{+}containing cells

S1 Fig. FACS analysis of Bone Marrow precursor-derived macrophages (BMF) and Dendritic cells (DC).
