# Supplementary figures and images for: Type 1 diabetes and parasite infection: An exploratory study in NOD mice
Source: PLoS One. 2024 Oct 22;19(10):e0308868. doi: 10.1371/journal.pone.0308868 (PMC11495574; doi:10.1371/journal.pone.0308868)

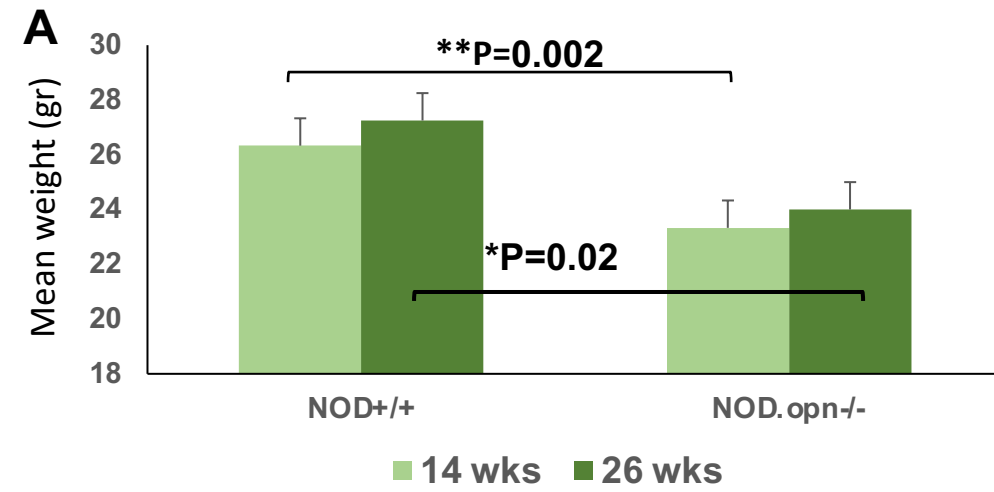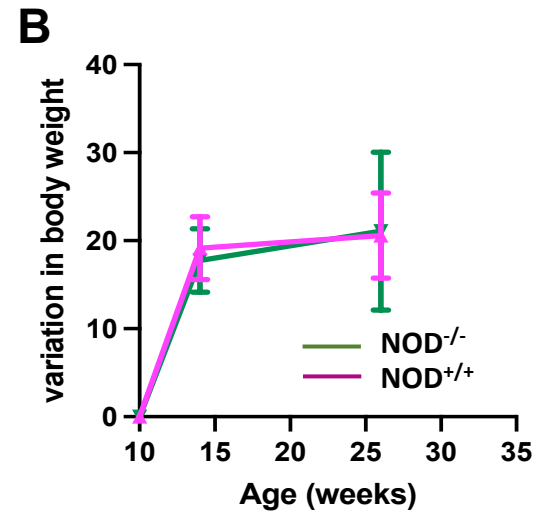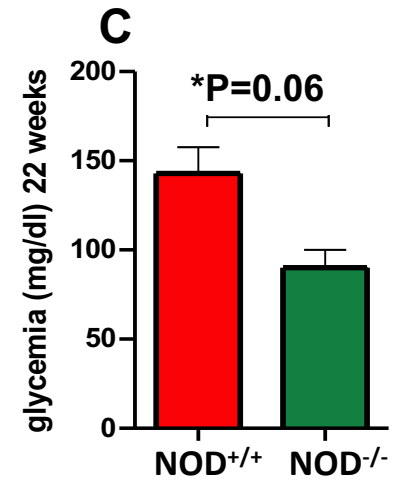

**S2 Fig. Physiology of infection.**

Supplement: S2 Fig — A. Mean weight (gr) of the NOD wild-type (NOD+/+) and opn knockout (NOD.opn-/-) mice after L. amazonensis infection. B. Post-infection variation of body weight, by the age of mice. C. Glycemia in infected with L. amazonensis NOD wild type and NOD opn knockout mice at 22 weeks of age. (PDF) [file pone.0308868.s005.pdf]

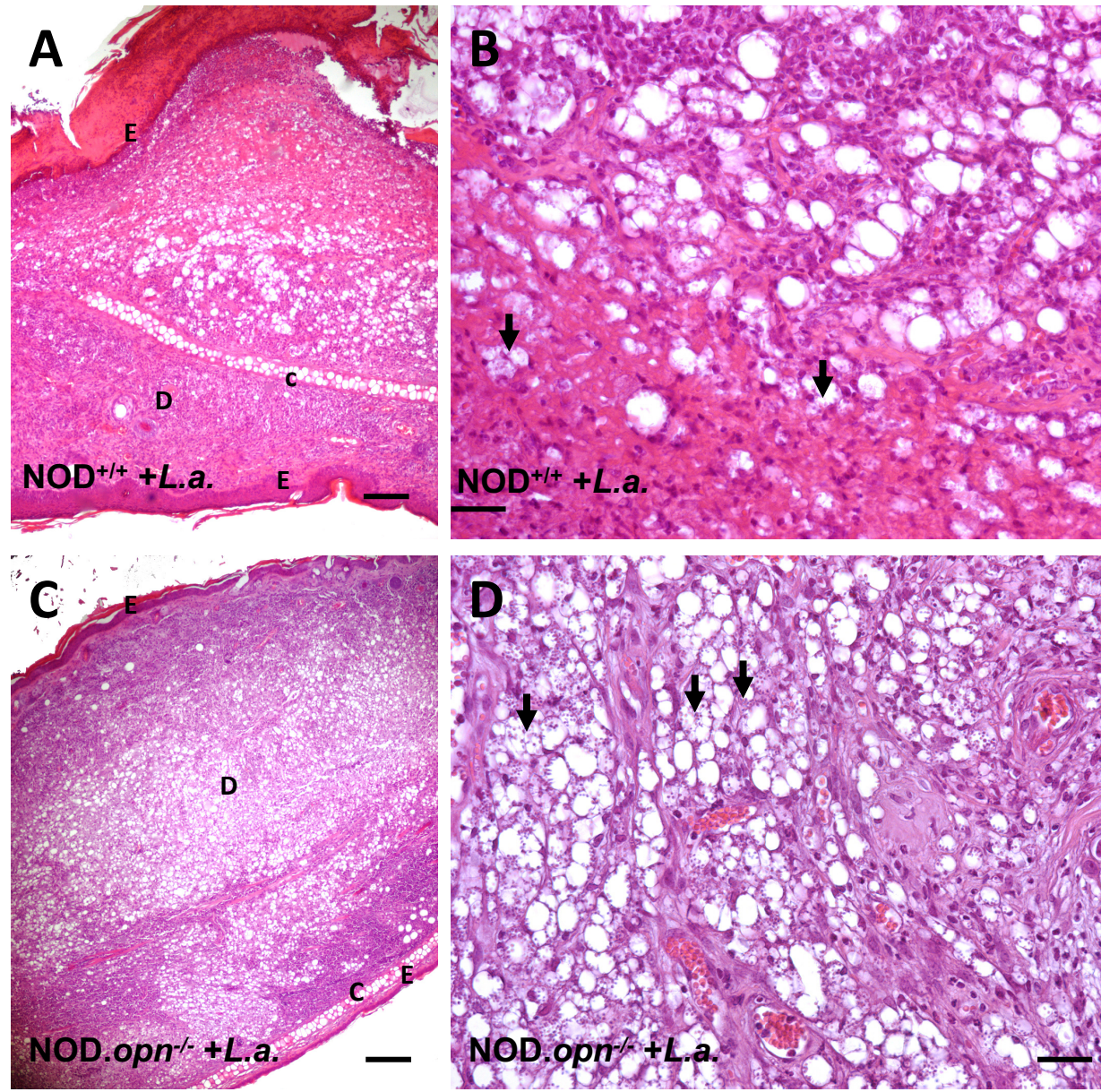

S3 Fig. Histological examination of the infected sites in the ear pinna.

Supplement: S3 Fig — A & B. Infected ear pinna from NOD wild-type mice (NOD+/+). C & D. Infected ear pinna from NOD.opn-/- mice. Hematoxylin and Eosin (H&E), A & C: Original Magnification x4, scale: 250, B & D, Original Magnification x4, scale bar: 50 μm. E: epidermis, D: dermis, C: cartilage. Arrows show the accumulation of parasites in the inflammatory cells. (PDF) [file pone.0308868.s006.pdf]

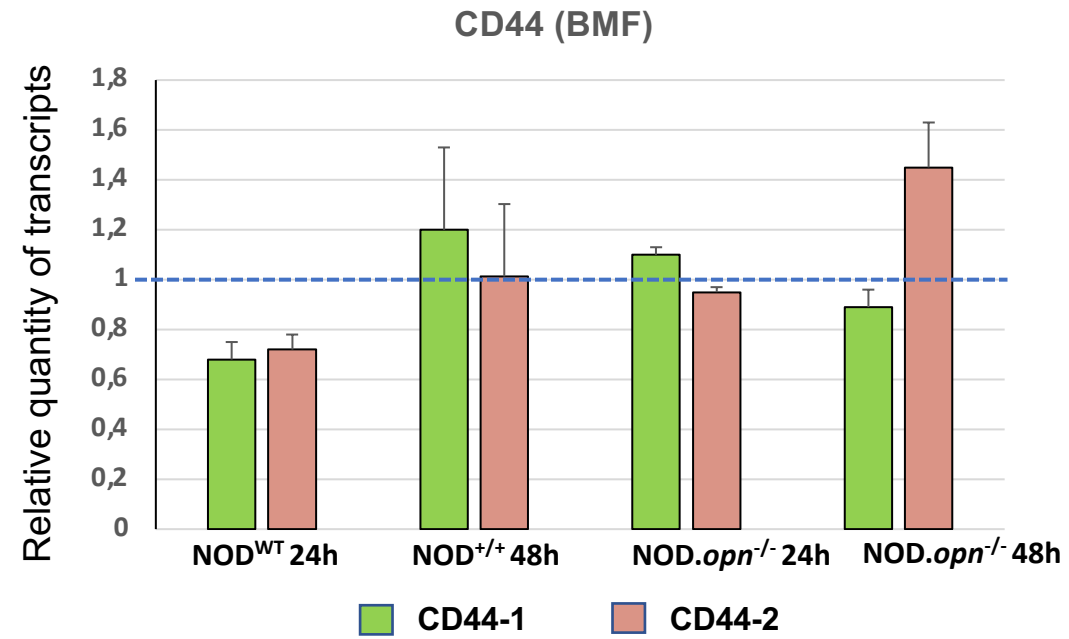

**S5 Fig. Real-time PCR for the OPN receptor CD44 in the BMF.**

Supplement: S5 Fig — Two sets of primers (CD44-1 and CD44-2) were used (see S3 Table). (PDF) [file pone.0308868.s008.pdf]

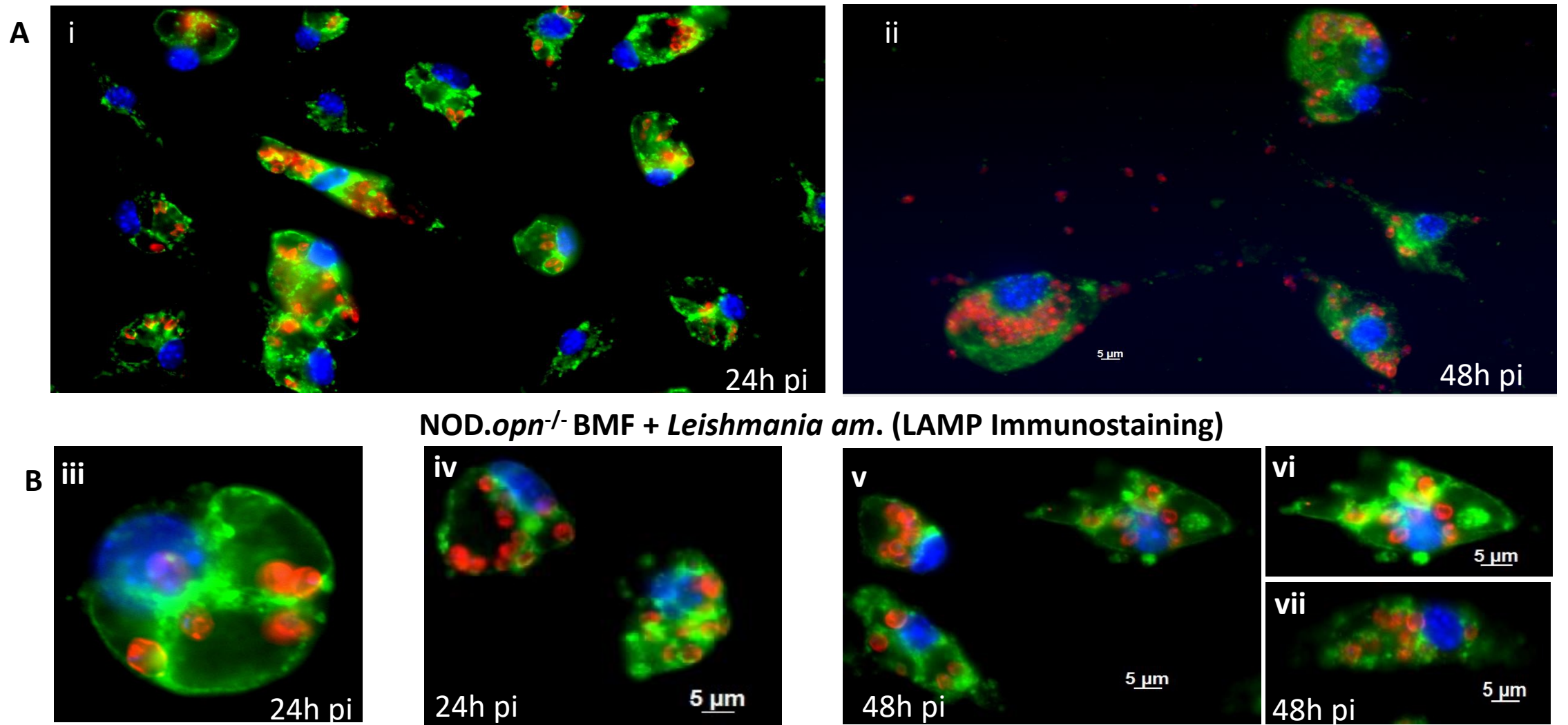

**S7 Fig. OPN favours parasite proliferation in the NOD genetic background (NOD<sup>+/+</sup>).**

Supplement: S7 Fig — BMF + Leishmania am. (LAMP Immunostaining). A. BMFs isolated from NOD wild-type (NOD/LtJ or NOD+/+) mice at 24h p.i. with L. am. (i) and at 48h p.i. (ii). B. BMFs isolated from NOD KO (NOD.opn-/-) mice at 24h p.i. (iii and iv) and at 48h p.i. (v, vi and vii). Pyroptosis-like cell swelling and membrane blending with intact nuclei and releasing of the parasites were observed only in the presence of OPN (ii). The numbers of cells examined are as in the legend of Fig 7. Parasite Numbers, crowding WT vs KO at 48h: 24.07 vs 8.76; P<0.05, CI 97.5%. Data and statistics are from the QP 3.0 program as described in S2 Table and M&M. (PDF) [file pone.0308868.s010.pdf]
