# Supplementary material for: Type 1 diabetes and parasite infection: An exploratory study in NOD mice
Source: PLoS One. 2024 Oct 22;19(10):e0308868. doi: 10.1371/journal.pone.0308868 (PMC11495574; doi:10.1371/journal.pone.0308868)
Supplement: S4 Fig — Representative experiment of BMF infected with Leishmania amazonensis, in the presence (NOD+/+) or in the absence (NOD.opn-/-) of osteopontin at 24h and 48h p.i. Total cell numbers are presented in S2 Table (QP 3,0 program for stats). NOD+/+ at 24h: 152 cells, NOD+/+ at 48h: 58 cells. NOD.opn-/- at 24h: 29 cells and for NOD-/- at 48h: 8 cells. (PDF) [file pone.0308868.s007.pdf]

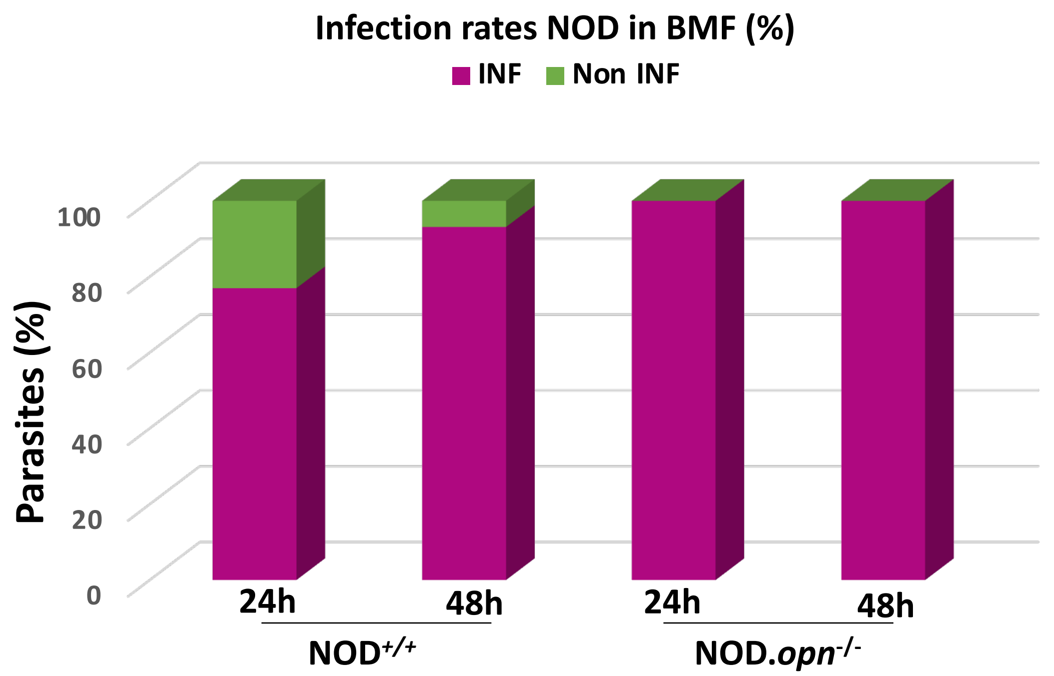

S4 Fig. Efficiency of cell transmission of *Leishmania amazonensis* parasites in bone marrow-derived macrophages (BMF).
