# Supplementary material for: Type 1 diabetes and parasite infection: An exploratory study in NOD mice
Source: PLoS One. 2024 Oct 22;19(10):e0308868. doi: 10.1371/journal.pone.0308868 (PMC11495574; doi:10.1371/journal.pone.0308868)
Supplement: S6 Fig — Relative quantities are expressed in ratios of infected (i) versus non-infected (non-inf) opn transcripts in the BMF at 24h post-infection. (PDF) [file pone.0308868.s009.pdf]

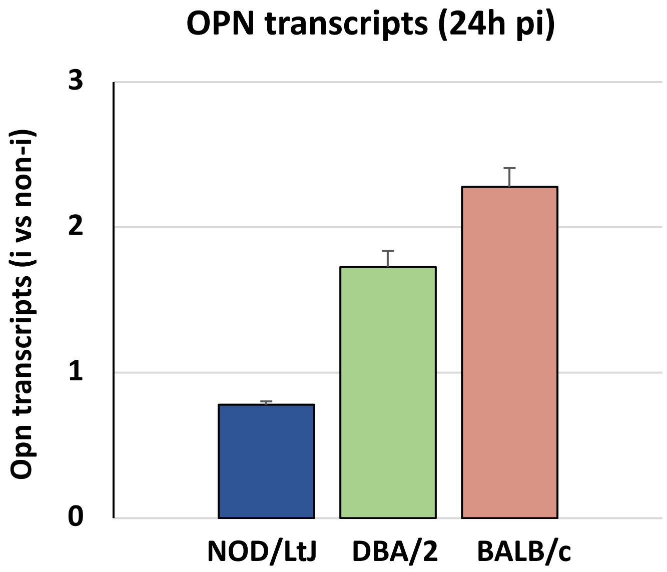


**S6 Fig. Comparison of the relative quantities of *opn* transcripts by q-RT-PCR of NOD/*LtJ*, DBA/2 and Balb/c strains of mice.**
